# 2,4-Dichlorophenoxyacetic Acid Poisoning Mimicking as Organophosphorus Poisoning

**DOI:** 10.7759/cureus.12852

**Published:** 2021-01-22

**Authors:** Ajithkumar Rajendran, Sasikumar Mahalingam, Guguloth Ramesh Babu, Kagne Rajeshwari Rajendra, Balamurugan Nathan

**Affiliations:** 1 Department of Emergency Medicine, Jawaharlal Institute of Postgraduate Medical Education and Research, Pondicherry, IND

**Keywords:** urinary alkalinization, dichlorophenoxyacetic acid poisoning, rhabdomyolysis, organophosphorus poisoning

## Abstract

Different compounds such as organochlorines, pyrethroids, fungicides, 2,4-dichlorophenoxy (2,4-D) herbicides, mushrooms, opioids, cartap compounds, and amitraz compounds can mimic organophosphorus (OP) poisoning. Muscle fasciculation, pulmonary edema, convulsions, bradycardia, hypotension, and smell caused by pyrethroids, as well as neurological signs, seizures, pulmonary edema, and smell caused by organochlorines can mimic OP poisoning. Miosis, vomiting, coma, and hypotension caused by opioids; miosis, bradycardia, altered sensorium, respiratory depression, and hypotension caused by amitraz compounds; and vomiting, breathlessness, altered sensorium, hypotension, and seizures caused by cartap compounds can also mimic OP poisoning. Mushroom poisoning and few fungicide compounds are also known to mimic features of OP poisoning. Hyperglycemia and glycosuria are the key hallmarks of amitraz poisoning. 2,4-D compounds can also mimic most of the features of OP poisoning; however, rhabdomyolysis, coma, and hyper/hypotonia are key differentiating features. Allergic manifestation and greenish discoloration of the contacted skin are the differentiating features of cartap poisoning.

Treating all agriculture-related poisoning with atropine without confirming the compound can lead to a therapeutic misadventure. Here, we discuss the case of a patient who was referred to our Emergency Department (ED) with an alleged history of an unknown poison ingestion which was managed with atropinization for suspected OP poisoning in an outside hospital. On probing the history, the actual compound was found to be a 2,4-D herbicide. Very few documented case reports of 2,4-D poisoning are available in the literature. Hyper/hypotonia, coma, and skeletal muscle damage are the key differentiating features of 2,4-D poisoning. Our patient had skeletal muscle damage (rhabdomyolysis), evidenced by raised creatine kinase-total and creatine kinase-muscle/brain. As there is no specific antidote, we treated the patient with urinary alkalinization and supportive care. The patient had a favorable outcome in the ED.

## Introduction

Chlorophenoxy compounds are used as an herbicide for the elimination of broadleaf plants, mainly within wheat, soybean, and corn crops. They are available as free acids, esters, amines, and salts. There are nine forms of chlorophenoxy compounds, including 2,4-dichlorophenoxyacetic acid, sodium salt, diethanolamine salt, dimethylamine salt, isopropylamine salt, triisopropanolamine salt, butoxy ethyl ester, ethyl hexyl ester, and isopropyl ester. 2,4-Dichlorophenoxy (2,4-D) poisoning is uncommon in our part of the country. Though 2,4-D poisoning can affect most organs, the kidney is the most sensitive target organ, and the classical features include gastrointestinal toxicity, myotoxicity, and neurotoxicity. There is no specific antidote for 2,4-D herbicide poisoning. Early initiation of urinary alkalinization (UA) can decrease the systemic toxicity and can also hamper the progression of rhabdomyolysis and acute renal failure. Emergency physicians should be aware of this poisoning, its clinical features, and treatment modalities, which can be lifesaving for patients. Here, we describe a case of 2,4-D poisoning, who developed rhabdomyolysis, neurotoxicity, and gastrointestinal toxicity but recovered to normal state due to UA and supportive care.

## Case presentation

A 65-year-old male presented to our Emergency Department (ED) with an alleged history of an unknown poison ingestion followed by multiple episodes of vomiting, diarrhea, excessive salivation, sweating, breathlessness, and muscle fasciculations. He was initially taken to a nearby hospital where he was treated with gastric lavage and atropine (bolus and infusion). The patient was then referred to our hospital for further management. On probing the history, it was found that the patient had consumed 50 mL of 2,4-D herbicide with an intent to commit suicide due to family issues.

On arrival to our ED, the patient was unconscious, pulse rate was 120/min, blood pressure was 110/70 mmHg, respiratory rate was 25/min, and oxygen saturation was 87% on room air. General physical examination, cardiovascular, and per abdomen examination were normal. Central nervous examination showed E2V2M5 Glasgow Coma Scale, dilated pupils, generalized hypertonia, hyperreflexia, ankle clonus, and muscle fasciculations. Right basal crepitations were present. Serum electrolytes, renal function test, liver function test, and blood sugar (104 mg/dL) were normal. Arterial blood gas (ABG) showed mild respiratory alkalosis. Electrocardiogram showed sinus tachycardia, and point-of-care echo showed an ejection fraction of 55% and normal chambers. Point-of-care ultrasound assessment of the inferior venacava showed a diameter of 1.5 cm and >50% collapsibility.

The patient was treated with supplemental oxygen, intravenous crystalloids, and UA. His sensorium gradually improved to E4V5M6 within 12 hours without any additional support. Creatine kinase-total (CK-T) and creatine kinase-muscle/brain (CK-MB) were elevated [maximum on day two: CK-T = 10259 IU/L (reference value, 0-171 IU/L); CK-MB = 133 IU/L (reference value, 0-24 IU/L)], but gradually CK values reduced and rhabdomyolysis settled without any need for dialysis.

## Discussion

Chlorophenoxy herbicide poisoning is not common, although it is a commonly available weed killer used in India. 2,4-D amine is the most commonly ingested form of chlorophenoxy herbicide poison. It is a fatal poison at higher doses that needs early diagnosis and prompt treatment. 2,4-D compounds are available in different concentrations (0.12%-96.9%). Human exposure occurs through ingestion, inhalation, skin absorption, or eye contact. Amine salt formulation can cause irreversible eye damage, whereas ester formulations are non-irritating to the eyes. The degree of dermal absorption may be lesser with amine salt formulations than with esters. Dermal exposure can cause skin irritation, chloracne, and sensory-motor peripheral neuropathy.

The minimum toxic dose of 2,4-D in humans is 3-4 g or 40-50 mg/kg, and death has been shown to occur after 6.5 g ingestion in adults [1,2]. In general, the acute lethal serum levels of 2,4-D appears to lie between 447 and 826 mg/L. Blood levels of 2,4-D can be measured most accurately using gas-liquid chromatography with electron-capture.

Toxicodynamics and toxicokinetics of 2,4-D

The production and degradation of 2,4-D lead to the creation of many compounds, including chlorophenols or dioxins, that cause severe toxicity. The probable mechanisms include mitochondrial injury, dose-dependent cell membrane damage, uncoupling of oxidative phosphorylation, disruption of acetyl coenzyme, and DNA damage/apoptosis by the free radical reaction [1,3]. 2,4-D causes cell apoptosis due to the change in membrane potential in mitochondria and initiates caspase-dependent reactions. It also affects mitochondrial electron transport system and causes reactive oxygen species (ROS) generation. This oxidative stress may affect the cells due to an imbalance between the production of potentially toxic ROS and physiological scavenging molecules. Oxidative stress causes lipid peroxidation, cytochrome c release from mitochondria, caspase-3 activation in affected cells, cytotoxic effect, and apoptosis. Moreover, increased lipid peroxidation can affect the activities of protective enzymatic antioxidants [3].

2,4-D is readily absorbed in the gastrointestinal and respiratory tract; however, dermal absorption is relatively low. 2,4-D is rapidly eliminated unchanged in the urine. The toxicokinetics of 2,4-D is dependent mainly on renal clearance. The differential capacity for excreting 2,4-D plays a vital role in the susceptibility to 2,4-D-induced effects.

Toxicity of 2,4-D

2,4-D toxicity can affect a wide range of organs ranging from the liver, kidneys, muscle, heart, lungs, gastrointestinal tract, nervous system (central, peripheral), endocrine system, and reproductive system [4]. Among them, gastrointestinal tract manifestation, skeletal muscle damage leading to rhabdomyolysis, and central nervous system depression leading to coma are the classical findings in 2,4-D toxicity (Table 1). On chronic exposure, 2,4-D can negatively affect the endocrine system (specifically the thyroid and gonads) and immune system.

**Table 1:** Classical effects of 2,4-dichlorophenoxyacetic acid poisoning. GI, gastrointestinal

Gastrointestinal toxicity	Musculotoxicity	Neurotoxicity
Corrosive effect causing throat pain, GI bleeding	Muscle fibrillation, Muscle fasciculation	Altered mental status (agitation, confusion, drowsiness, coma)
Abdominal pain, vomiting	Severe myalgia	Seizure
Diarrhea	Rhabdomyolysis	Ataxia
	Myopathy	Nystagmus
	Myotonia, muscle rigidity	

Gastrointestinal toxicity, myotoxicity, and neurotoxicity are the classical features of 2,4-D poisoning (Table 1). It causes neuromuscular toxicity and myotonia by inhibiting voltage-gated chloride channels [5]. At higher doses, blood-brain barrier disruption occurs, leading to neurotoxicity. Musculotoxicity usually manifests as rhabdomyolysis leading to myoglobin excretion in urine. Disruption of the cellular tubulin-microtubule network is one of the key mechanisms in the induction of lung cytotoxicity, manifesting as pulmonary hemorrhage, pulmonary edema, and tracheobronchial congestion [6]. Cardiovascular effects include toxic myocarditis and cardiac arrhythmias (atrial fibrillation, bradyarrhythmia).

2,4-D toxicity can cause refractory hypotension, which can be due to gastrointestinal loss, loss of vascular resistance, or direct myocardial toxicity. Bradberry et al. found that cases with coexisting coma and hypotension had a poor prognosis [4,7]. Other features include pyrexia, miosis, chromodacryorrhea, hyperglycemia, hyperkalemia, hypokalemia, hypocalcemia, metabolic acidosis, and peripheral neuropathy. Massive rhabdomyolysis, metabolic acidosis, and severe and intractable hypotension have also been reported, resulting in death within 24 hours. Renal failure (tubular necrosis, rhabdomyolysis-induced acute kidney injury) [8], toxic liver injury (aspartate aminotransferase and alanine transaminase can be elevated), neurotoxic effects (ataxia, hypertonia, seizures, and coma) may also occur.

Role of UA in 2,4-D poisoning

A UA regimen increases poison elimination by administering intravenous (IV) sodium bicarbonate to produce urine with a pH of ≥7.5. UA can be used for different poisonings, namely, 2-4-D, chlorpropamide, salicylate, barbiturates, methotrexate, diflunisal, fluoride, and mecoprop [9]. Ionized substances have a decreased rate of diffusion from renal tubules back into the circulation and are eliminated more rapidly. Acidic drugs get ionized in an alkaline environment, which enhances their renal excretion. 2-4-D are weak acids that are eliminated unchanged in the urine. Its renal clearance is directly proportional to urinary pH. Hence, UA technique by ion trapping can help enhance its elimination, thereby reducing neurotoxic and myotoxic features. Plasma alkalinization may also limit the distribution of phenoxy compounds from the central circulation by ion trapping [8,10,11]. The impact of UA depends upon the extent and persistence of the pH change. UA (pH > 7.5) with high urine flow rate (200-600 mL/hour) had renal clearance almost equal to that of dialysis [7,9,10].

Before starting UA, IV fluid resuscitation (if any fluid deficit), Foley placement, baseline investigation, namely, electrolyte, blood urea, creatinine, ABG, urine pH (fresh urine), blood sugars, and serum toxin levels, should be done. Steps of UA include correction of preexisting hypokalemia if present, 1-2 mEq/kg of 8.4% bicarbonate IV bolus over 5-10 minutes followed by 100-150 mEq 8.4% bicarbonate in 1 L of 5% dextrose in water (d5w) or 5% dextrose at 200-250 mL/hour IV infusion, injection KCl 20-40 mEq/L to maintain normokalemia and four-hourly serum potassium and bicarbonate monitoring [12,13].

Aim for urinary pH goal of 7.5-8.5, ABG pH of <7.55-7.60, and urine output of 100-200 mL/hour. The complications expected are severe alkalemia, hypokalemia, alkalotic tetany, hypocalcemia, coronary vasoconstriction, and cerebral vasoconstriction. Our patient was strictly monitored during UA and did not present any of these complications. Established or incipient renal failure is a contraindication to UA. Significant preexisting heart disease is a relative contraindication.

Role of enhanced diuresis in UA in 2,4-D poisoning

Forced diuresis enhances poison elimination by reducing its concentration in the renal tubular fluid and, therefore, the gradient for reabsorption. The same result may be achieved by increasing the rate of flow of filtrate in the nephron and reducing the time spent in the tubule. Reabsorption can be further reduced and elimination can be enhanced by trapping the poison in the urine (ion trapping) by manipulating urine pH in such a manner so as to keep it in an ionized state [9].

Hemodialysis in 2,4-D poisoning

Durakovic et al. recommended hemodialysis in the early phase of high-dose 2-4-D poisoning [1]. Low molecular weight, low volume of distribution, high water solubility, and low renal clearance make hemodialysis suitable [1]. However, extensive protein binding can hinder the efficacy of hemodialysis [7,14].

The patient was treated with UA, following which he improved dramatically. Early initiation of UA may prevent acute kidney injury, systemic toxicity, thereby preventing the need for dialysis, improving the patient’s sensorium, and avoiding intubation. The management is mainly supportive as no specific antidote is available. In severe toxicity, hemoperfusion, hemodialysis, and plasmapheresis can be used [1,7]. UA with high-flow urine output enhances herbicide elimination and can be considered in seriously ill patients.

## Conclusions

The classical systemic toxicity of 2,4-D poisoning is gastrointestinal toxicity, myotoxicity, and neurotoxicity, which can be effectively managed with UA. The key features that can be used to differentiate 2,4-D poisoning from organophosphorus poisoning are hyper/hypotonia, coma, and rhabdomyolysis. It is always better to confirm the compound than treating all agriculture-related poisoning with atropine inappropriately.
